# Towards a unified scheme of cortical lamination for primary visual cortex across primates: insights from NeuN and VGLUT2 immunoreactivity

**DOI:** 10.3389/fnana.2014.00081

**Published:** 2014-08-15

**Authors:** Pooja Balaram, Jon H. Kaas

**Affiliations:** Laboratory of Jon Kaas, Department of Psychology, Vanderbilt UniversityNashville, TN, USA

**Keywords:** visual cortex, primate, tree shrew, NeuN, cortical layers, Brodmann, Hässler

## Abstract

Primary visual cortex (V1) is clearly distinguishable from other cortical areas by its distinctive pattern of neocortical lamination across mammalian species. In some mammals, primates in particular, the layers of V1 are further divided into a number of sublayers based on their anatomical and functional characteristics. While these sublayers are easily recognizable across a range of primates, the exact number of divisions in each layer and their relative position within the depth of V1 has been inconsistently reported, largely due to conflicting schemes of nomenclature for the V1 layers. This conflict centers on the definition of layer 4 in primate V1, and the subdivisions of layer 4 that can be consistently identified across primate species. Brodmann’s (1909) laminar scheme for V1 delineates three subdivisions of layer 4 in primates, based on cellular morphology and geniculate inputs in anthropoid monkeys. In contrast, Hässler’s (1967) laminar scheme delineates a single layer 4 and multiple subdivisions of layer 3, based on comparisons of V1 lamination across the primate lineage. In order to clarify laminar divisions in primate visual cortex, we performed NeuN and VGLUT2 immunohistochemistry in V1 of chimpanzees, Old World macaque monkeys, New World squirrel, owl, and marmoset monkeys, prosimian galagos and mouse lemurs, and non-primate, but highly visual, tree shrews. By comparing the laminar divisions identified by each method across species, we find that Hässler’s (1967) laminar scheme for V1 provides a more consistent representation of neocortical layers across all primates, including humans, and facilitates comparisons of V1 lamination with non-primate species. These findings, along with many others, support the consistent use of Hässler’s laminar scheme in V1 research.

## INTRODUCTION

Primary visual cortex, or V1, in most mammalian species is clearly distinguishable from other cortical areas by the crisp, stratified appearance of its cortical layers (Campbell, 1905; Brodmann, 1909; Hässler, 1967; Garey, 1971; Billings-Gagliardi et al., 1974; Braak, 1984; Jones, 1984; Le Brun Kemper and Galaburda, 1984). In primates, including humans (Allman and Kaas, 1971; Lund, 1973; Braak, 1976; Fitzpatrick et al., 1983; Preuss et al., 1993; Casagrande and Kaas, 1994; de Sousa et al., 2010; Wong and Kaas, 2010), as well as some related non-primate species (Collins et al., 2005; Wong and Kaas, 2008; Wong and Kaas, 2009b), most of these layers have expanded and segregated into sublayers with distinct functional and connectional properties, making V1 even more conspicuous in comparison to other cortical areas. Yet despite its marked appearance and the clear identification of multiple layers and sublayers across species, the classification of cortical layers within V1 of primates remains a controversial topic (Campbell, 1905; Clark and Sunderland, 1939; von Bonin, 1942; Garey, 1971; Billings-Gagliardi et al., 1974; Henry, 1989; Casagrande and Kaas, 1994; Kaas, 2003; Zilles and Amunts, 2010; Molnár and Belgard, 2012; Nieuwenhuys, 2013). Numerous classification systems have been proposed over the history of V1 research; some based on laminar variations in staining intensity for histological markers such as cytochrome oxidase (CO; Livingstone and Hubel, 1982; Horton, 1984), some based on changes in cell density and morphology through the cortical sheet, as seen with Nissl, myelin, or Golgi stains (Lewis, 1880; Cajal, 1899; Campbell, 1905; Brodmann, 1909; Clark, 1925; von Economo and Koskinas, 1925; von Bonin, 1942; Hässler, 1967; Garey, 1971; Valverde, 1977), and still others based on variations in layer-specific gene and protein expression patterns through the areal extent of V1 (Hevner et al., 2003; Watakabe et al., 2006; Yamamori and Rockland, 2006; Bernard et al., 2012; Bryant et al., 2012; Takahata et al., 2012). Within a single primate species such as macaque monkeys, which are a widely utilized primate model for V1 research, anywhere from eight to twelve layers and sublayers can be identified depending on the criteria for classification (Lund, 1973; Billings-Gagliardi et al., 1974; Felleman and Van Essen, 1991; Casagrande and Kaas, 1994), leading to enormous confusion when comparing studies of layer-specific connections or functions in V1. In less specialized primates such as prosimians or lemurs (Preuss et al., 1993; Preuss and Kaas, 1996; Wong and Kaas, 2009a; Wong et al., 2009), where cortical layers are less distinct, only six to eight layers are commonly identified in anatomical studies, making the comparison of laminar-specific properties across primate species rather difficult. Thus, a common system of laminar classification across primates would greatly benefit the interpretation of past and future studies of V1’s structure and function. Of course this laminar system should be consistent with laminar patterns used in other cortical regions in primates, and for primary visual cortex in non-primate taxa.

The most common discrepancy seen between laminar schemes for V1 in primates involves the superficial and middle cortical layers. Under the famous scheme of Brodmann (1909), layer 3 of V1 is a single layer and layer 4 is divided into three distinct sublayers, termed 4A, 4B, and 4C, with 4C further divided into 4Cα and 4Cβ. This definition, originally based on Nissl-stained sections through the border of V1 with the second visual area (V2) in anthropoid primates and humans, stemmed from the observation that all three middle layers merged into a single layer 4 in V2 and thus, must all be derived from a single layer 4 in ancestral primates. Another dominant laminar scheme, that of Hässler (1967), suggests that layer 3 of V1 is expanded into three layers, termed 3A, 3B, and 3C, and layer 4 only contains two subdivisions, 4A and 4B. When compared directly, Brodmann’s 4A, 4B, 4Cα, and 4Cβ are Hässler’s 3Bβ, 3C, 4A, and 4B, respectively, which leads to significant confusion when discussing the origin and function of layers 3 and 4 in primate V1.

Anatomical studies of V1 in mammalian brains have benefited from recent advances in histology, immunohistochemistry, and in situ hybridization techniques that allow for the selective labeling of individual cell populations based on cell- or layer-specific markers (Hevner et al., 2003; Yamamori and Rockland, 2006; Belgard et al., 2011; Yamamori, 2011; Bernard et al., 2012; Molnár and Belgard, 2012; Takahata et al., 2012). Many markers can be variably expressed across layers, within and across species, so converging types of evidence from different markers is more informative (Molnár and Belgard, 2012), but some of them do distinguish the same layers of V1 across multiple species. Thus, a reconsideration of laminar classification in V1 of primates is timely, given the benefits of reliable layer-specific markers and the vast array of studies on layer-specific V1 functions in current research.

Two markers have gained widespread use in recent research by providing new insights on areal, laminar, and cellular functions within V1. The first is neuronal nuclear antigen (NeuN; Wolf et al., 1996), a DNA-binding protein that is exclusively expressed in neuronal cell bodies across the central nervous system (CNS) and highly conserved across a range of mammalian and non-mammalian species. Immunolabeling for NeuN distinguishes neurons from astrocytes, microglia, and endothelial cells, and identifies morphologically distinct classes of neurons in cortical and subcortical brain structures. Thus, NeuN resembles a Nissl stain but avoids the complications of labeling cells other than neurons. The second is vesicular glutamate transporter 2 (VGLUT2), a neurotransmitter transport protein found on the vesicles of many glutamatergic neurons in the CNS (Aihara et al., 2000; Hisano et al., 2000; Herzog et al., 2001). VGLUT2 is expressed in the presynaptic terminals of most thalamic relay neurons that project to primary sensory cortical areas (Kaneko et al., 2002), and immunolabeling for VGLUT2 reliably distinguishes terminal projections from the lateral geniculate nucleus (LGN) to the granular layer 4 of V1. (Balaram et al., 2011, 2013; Bryant et al., 2012; Garcia-Marin et al., 2012; Rovó et al., 2012). When combined with traditional markers such as CO and Nissl, both NeuN and VGLUT2 can provide additional information about anatomically and functionally distinct layers within V1.

In an attempt to define homologous V1 layers, we labeled NeuN and VGLUT2 through the cortical sheet of seven primate species (chimpanzees, macaque monkeys, owl monkeys, squirrel monkeys, marmoset monkeys, galagos, and mouse lemurs), and one highly visual close relative of primates (tree shrews). By then comparing NeuN- and VGLUT2-labeled sections to adjacent CO- and Nissl-labeled sections, we outlined laminar boundaries through V1 based on changes in the density and morphology of NeuN-labeled cells, as well as the discrete boundaries of VGLUT2-labeled geniculostriate terminations in each species. NeuN labeling revealed highly conserved cortical lamination patterns in V1 of every species examined in this study, and a clear visualization of specialized and homologous V1 layers, particularly at the border of V1 with extrastriate visual area, V2. Additionally, VGLUT2 labeling provided consistent demarcations of layer 4 in V1 across species, and identified specialized geniculostriate terminations to discrete sublayers of layer 3 and layer 6 in some primates. When compared across the primate lineage, both NeuN and VGLUT2 distributions provided evidence for multiple subdivisions of the superficial and deep V1 layers, but only one granular layer 4 with two subdivisions in V1. These layers are consistent with Hässler’s (1967) laminar scheme for V1. The laminar boundaries identified in both NeuN and VGLUT2 preparations are comparable to laminar divisions in other cortical areas across primates, as well as laminar divisions within V1 in non-primates, and may highlight specializations in V1 that reflect ethological and behavioral differences between primate groups.

## MATERIALS AND METHODS

NeuN and VGLUT2 immunoreactivity was examined in V1 of seven primate and one non-primate species: chimpanzees *(Pan troglodytes)*, macaques *(Macaca mulatta)*, marmosets *(Callithrix jacchus)*, squirrel monkeys *(Saimiri sciureus),* owl monkeys *(Aotus trivirgatus)*, galagos *(Otolemur garnetti)*, mouse lemurs *(Microcebus murinus),* and tree shrews *(Tupaia glis)*. At least two, and up to five, individual cases for each species were utilized in this study. All procedures involving mouse lemurs followed the guidelines established by the European Communities Council directives. All procedures involving chimpanzees, macaque monkeys, squirrel monkeys, owl monkeys, marmosets, galagos, and tree shrews followed the animal care and use guidelines established by the National Institutes of Health.

### TISSUE ACQUISITION AND HISTOLOGY

One chimpanzee brain was obtained through the tissue donation program at the Texas Biomedical Research Institute (San Antonio, TX, USA), from a 53-year-old female that had recently died of unrelated natural causes. Once the animal was pronounced naturally deceased by veterinary staff, the brain was flushed postmortem with 0.1 M phosphate buffered saline (PBS) and shipped overnight to Vanderbilt University. Upon arrival, the brain was bisected through the corpus callosum and subcortical structures, and one hemisphere was postfixed in 4% paraformaldehyde (PFA) for 2 days. Following postfixation, the hemisphere was blocked into smaller pieces to facilitate sectioning through V1, and cryoprotected in 30% sucrose in 0.1 M phosphate buffer (PB) for 24 h. The other hemisphere was preserved for use in related studies. Another chimpanzee brain was obtained from a deceased adult male at the New Iberia Research Center (Lafayette, LA, USA) several years prior to this study. This individual had also perished from natural causes, and once pronounced dead by a veterinarian, the brain was extracted from the skull and briefly rinsed in 0.1M PBS prior to postfixation with 4% PFA in 0.1M PBS. Following postfixation, the occipital lobe was blocked for coronal sections and cryoprotected in 30% sucrose in 0.1 M PB for 48 h prior to histology.

Two mouse lemur brains were obtained for this study from collaborators at INSERM in Bron, France, where the use of mouse lemurs for research purposes is supported under the Comité Consultatif National d’Éthique (CCNE). Both individuals were euthanized in France and transcardially perfused with 0.1 M PBS followed by 4% PFA in 0.1M PBS. One brain was extracted whole and shipped in 0.1 M PBS overnight to Vanderbilt University. This brain was blocked and cryoprotected in 30% sucrose for 24 h prior to histology. The other brain was extracted, cryoprotected in 20% glycerol in 0.1 M PBS and cut into 40 μm coronal sections, and the sections were shipped overnight to Vanderbilt University. These sections were then stored in 0.1 M Tris-buffered saline (TBS) with 0.1% sodium azide for immunohistochemistry.

Two macaque monkey brains were utilized in this study. One brain was obtained through the tissue donation program at the University of Washington (Seattle, WA, USA) and one brain was obtained from collaborators at Vanderbilt University. Following lethal levels of anesthesia, both individuals were euthanized and then transcardially perfused with 0.1 M PBS followed by 4% PFA in 0.1 M PBS. The brains were extracted from the skull and postfixed in 4% PFA for 3–6 h, and individual hemispheres were then blocked and cryoprotected in 30% sucrose for 24 h prior to histology.

Three squirrel monkeys, five owl monkeys, and four galagos were utilized in this study, all of which were obtained within Vanderbilt University. Following lethal levels of anesthesia, all individuals were transcardially perfused with 0.1M phosphate-buffered saline (PBS) followed by 4% PFA in 0.1 M PBS. Brains were extracted from the skull, blocked into appropriately sized pieces for sectioning, and cryoprotected in 30% sucrose in 0.1 M phosphate buffer for 24–72 h prior to histology.

Four tree shrews were obtained from collaborators at the University of Kentucky, Louisville for this study. All individuals were euthanized and transcardially perfused with 0.1 M PBS followed by 4% PFA in 0.1 M PBS. Brains were extracted from the skull, rinsed in 0.1 M PBS, and transported in 4% PFA to Vanderbilt University within 24 h. Upon arrival, brains were blocked into appropriately sized pieces for sectioning, and cryoprotected in 30% sucrose in 0.1 M phosphate buffer for 24 h prior to histology.

Cryoprotected blocks from each individual were sectioned into 40–50 μm coronal sections using a sliding microtome and separated into 5–10 alternating series, depending on the overall size of the brain. At least one series in every specimen was processed for CO (Wong-Riley, 1979) or Nissl with thionin to identify the boundary of V1 in each case. Remaining series were stored at 4°C in 0.1 M TBS with 0.1% sodium azide until further use.

### IMMUNOHISTOCHEMISTRY

One series in every specimen was labeled for NeuN, and a second series was labeled for VGLUT2, using standard immunohistochemical techniques (Balaram et al., 2013). Sections were rinsed twice in 0.01M PBS, postfixed in 2% PFA for 20 min, rinsed twice again, and incubated for 20 min in 0.01% hydrogen peroxide to quench background reactivity. Sections were then rinsed and incubated for 2 h in a blocking solution containing 5% normal horse serum (Sigma Aldrich, St. Louis, MO, USA) and 0.05% Triton-X100 (Acros Pharma, Princeton, NJ, USA) in 0.01 M PBS. Sections were then transferred to fresh blocking solution containing a 1:5000 dilution of either mouse anti-NeuN or mouse anti-VGLUT2 (both from Millipore, Billerica, MA, USA) primary antibody, and incubated at room temperature overnight. The following day, sections were rinsed thoroughly and incubated for 2 h in fresh blocking solution containing a 1:500 dilution of biotinylated horse anti- mouse IgG (Vector labs, Burlingame, CA, USA), then rinsed again and incubated overnight in a biotin amplification solution (ABC Elite kit, Vector Labs, Burlingame, CA, USA). After that, sections were rinsed thoroughly again and reacted with a solution containing 1% diaminobenzidine, 1% nickel chloride, and 0.1% hydrogen peroxide in 0.01 M PBS to visualize the label. All sections were then mounted on gelatin-subbed slides, dehydrated in ethanol, cleared in xylene, and coverslipped with Permount (Fisher Scientific, Pittsburgh, PA, USA).

### IMAGING AND ANALYSIS

**FIGURE 1:**
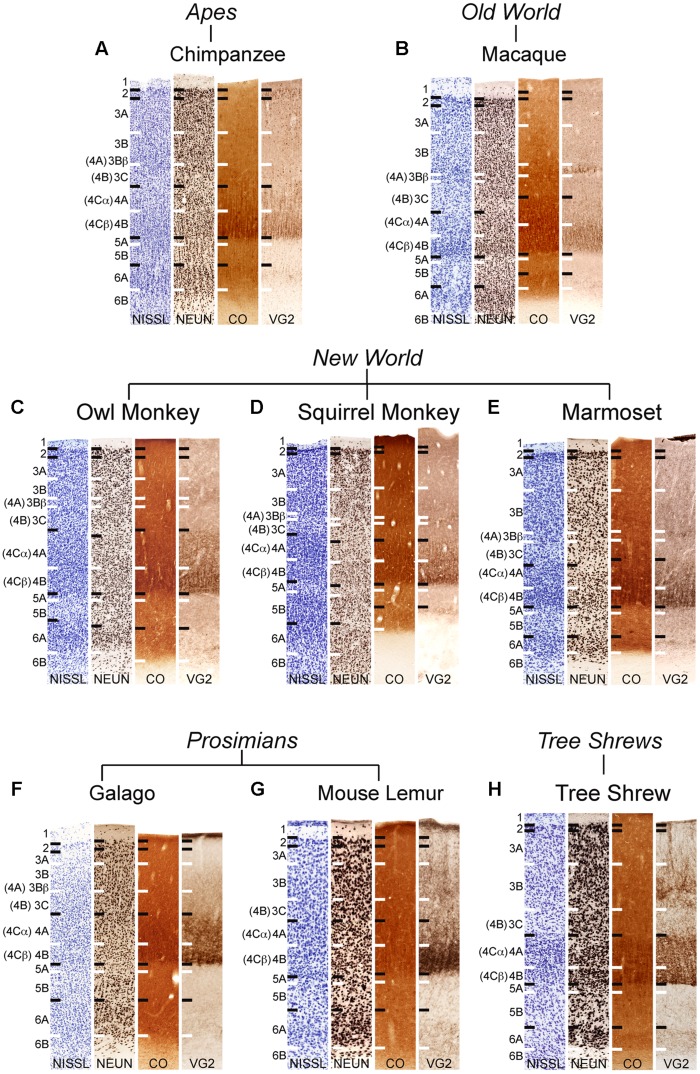
**Laminar distributions of Nissl, neuronal nuclear antigen (NeuN), cytochrome oxidase (CO), and vesicular glutamate transporter 2 (VGLUT2) in primary visual cortex of chimpanzees (A), Old World macaque monkeys (B), owl monkeys (C), New World squirrel monkeys (D), and marmosets (E), prosimian galagos (F) and mouse lemurs (G), and tree shrews (H)**. Hässler’s (1967) laminar divisions listed to the left, Brodmann’s (1909) divisions are listed in parentheses. Individual panels are scaled relative to V1 of chimpanzees to visualize comparisons between laminar density and staining intensity across primate species. Absolute scale comparisons are shown in **Figure 2**.

**FIGURE 2:**
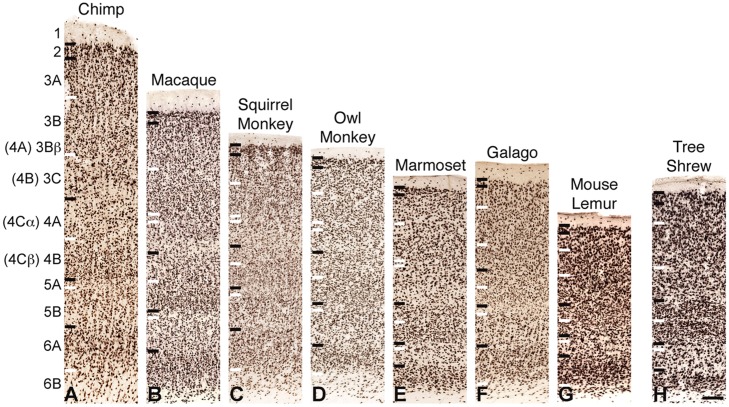
**NeuN immunoreactivity through the cortical layers of V1 in (A) chimpanzees, (B) macaque monkeys, (C) squirrel monkeys, (D) owl monkeys, (E) marmosets, (F) galagos, (G) mouse lemurs, and (H) tree shrews**. Laminar designations by Hässler (1967) are listed to the left, Brodmann’s divisions are listed in parentheses. Black lines indicate neocortical layer boundaries and white lines indicate sublaminar boundaries within each layer. Scale bar is 250um.

**FIGURE 3:**
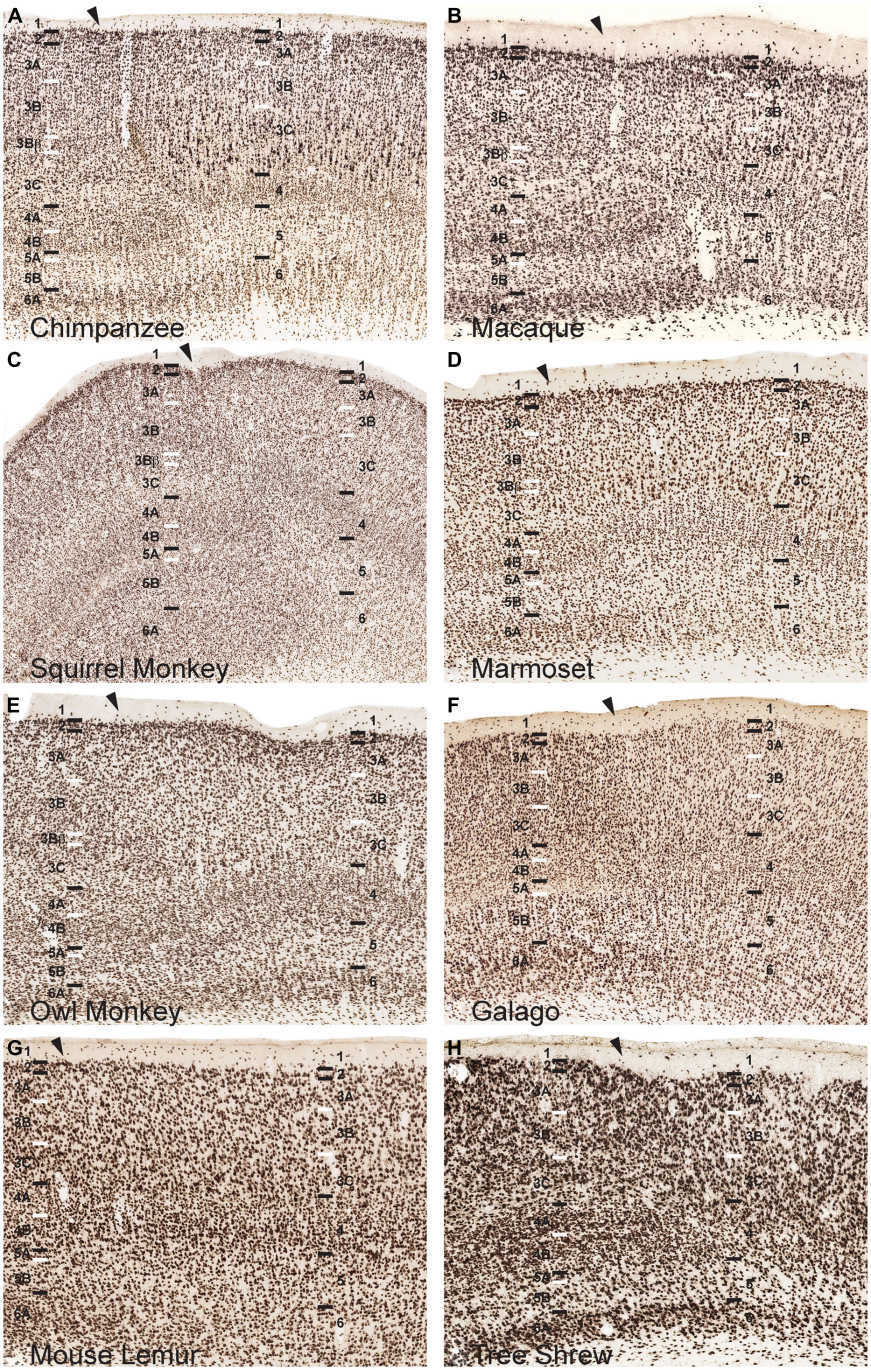
**NeuN immunoreactivity reveals transitions of laminar boundaries between V1 and V2 in (A) chimpanzees, (B) macaque monkeys, (C) squirrel monkeys, (D) marmosets, (E) owl monkeys, (F) galagos, (G) mouse lemurs, and (H) tree shrews**. Hässler’s laminar designations are listed on each panel for both visual areas. Arrowheads demarcate the V1/V2 border, V1 is to the left of the arrowhead and V2 is to the right of the arrowhead in each panel. Black lines indicate laminar boundaries and white lines indicate sublaminar boundaries in each area. Individual panels are scaled relative to V1 of chimpanzees to visualize laminar transitions between V1 and V2 across species.

**FIGURE 4:**
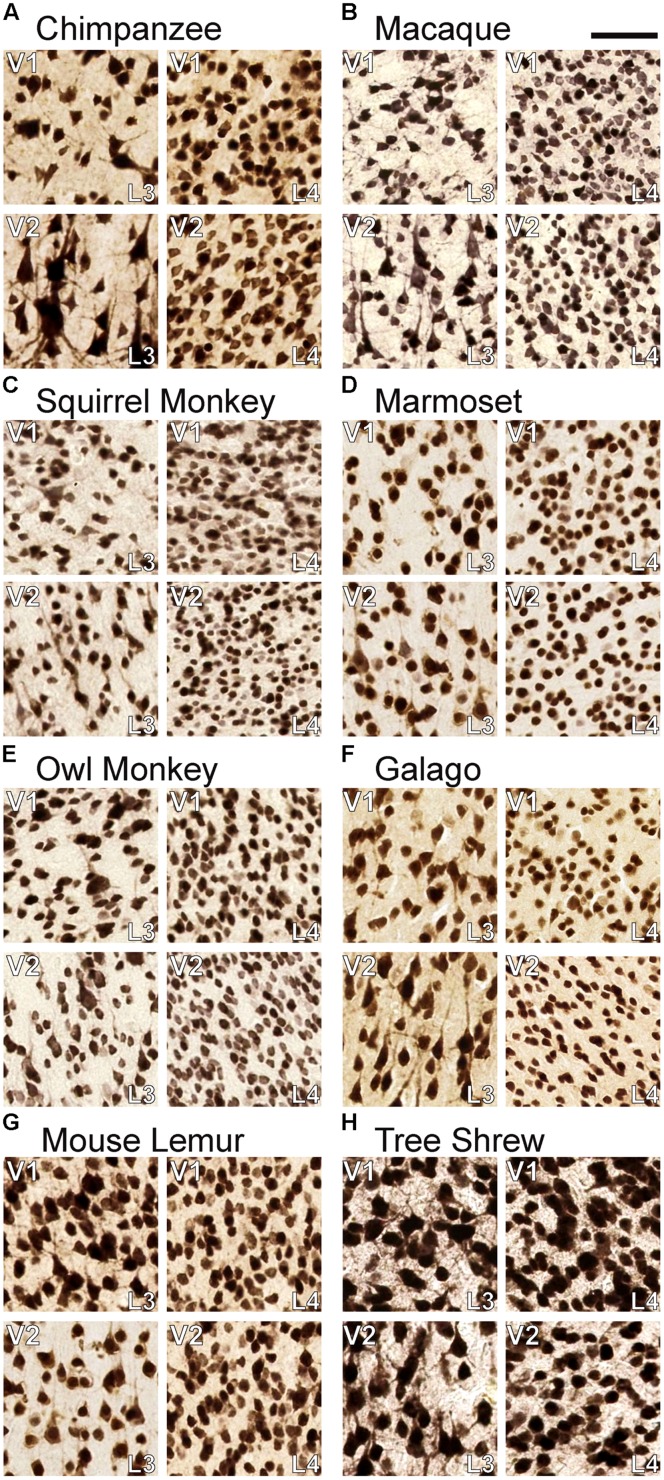
**Comparisons of neuronal density in layers 3C and 4 of (A) chimpanzees, (B) macaque monkeys, (C) squirrel monkeys, (D) marmosets, (E) owl monkeys, (F) galagos, (G) mouse lemurs, and (H) tree shrews**. In both areas, neurons in layer 4 are significantly more densely packed than neurons in layer 3C. Scale bar is 20 μm.

**FIGURE 5:**
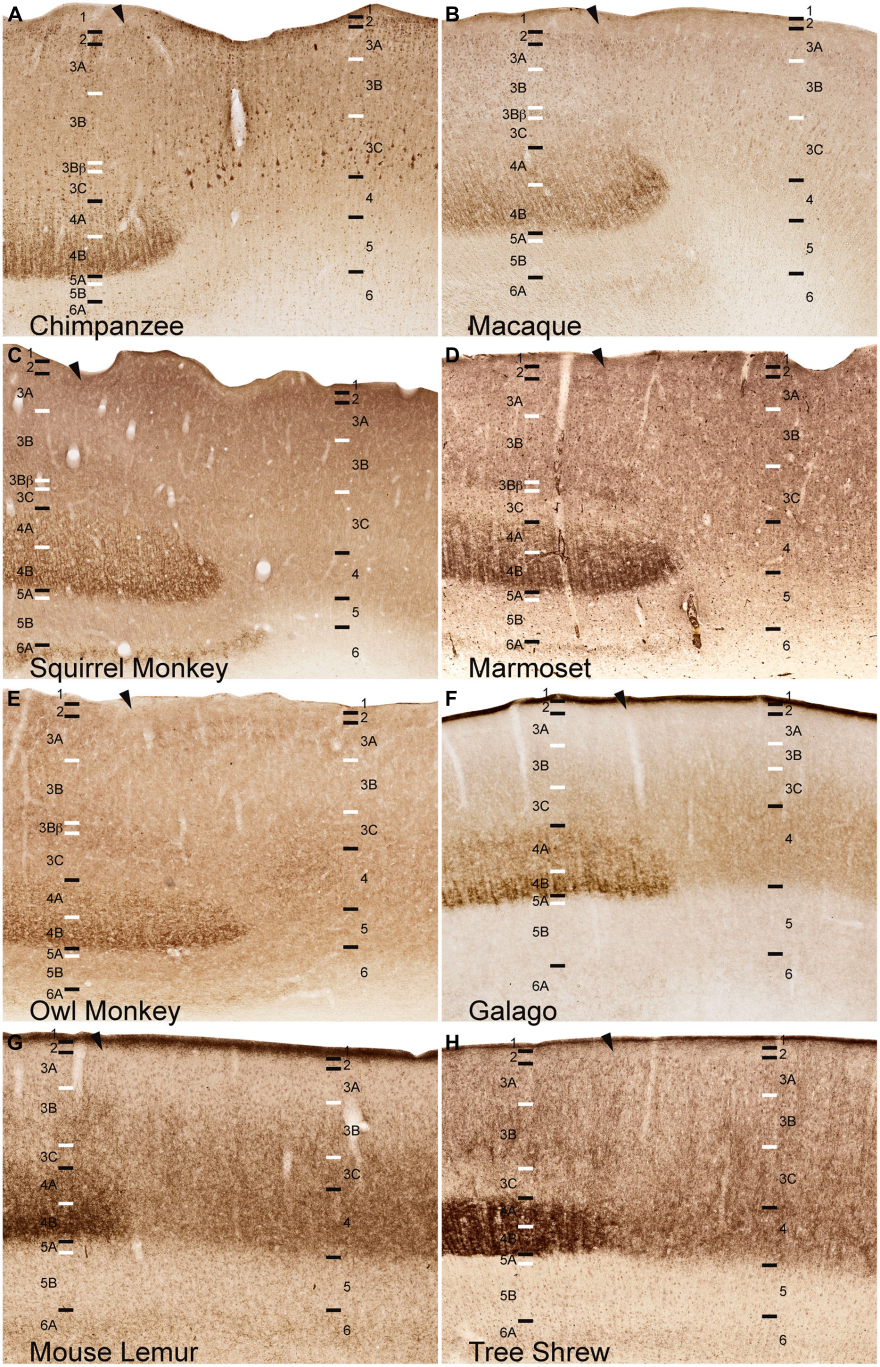
**VGLUT2 immunoreactivity reveals the continuation of layer 4, but not layer 3Bβ, at the boundary of V1 into V2 across (A) chimpanzees, (B) macaque monkeys, (C) squirrel monkeys, (D) marmosets, (E) owl monkeys, (F) galagos, (G) mouse lemurs, and (H) tree shrews**. Hässler’s laminar designations are listed on each panel for both visual areas. Arrowheads demarcate the V1/V2 border, V1 is to the left of the arrowhead and V2 is to the right of the arrowhead in each panel. Black lines indicate laminar boundaries and white lines indicate sublaminar boundaries in each area. Individual panels are scaled relative to V1 of chimpanzees to visualize laminar transitions between V1 and V2 across species.

All sections were imaged at 20× magnification using a Leica SCN400 slide scanner and individual images were exported using Leica Ariol software (Leica Microsystems, Buffalo Grove, IL, USA). All images were adjusted for brightness and contrast, but were otherwise unaltered for the purposes of this study. For **Figures 1, 3**, and **4**, images were resized relative to the largest panel (usually those depicting chimpanzee V1) to provide better comparisons and distinctions between laminar boundaries across the range of variably sized primates and tree shrews. For **Figures 2** and **5**, all images were held at absolute size to display the relative thickness of V1 layers compared to total cortical thickness in **Figure 2**, as well as the relative neuronal densities between layers of V1 and V2 in **Figure 5**.

For comparative estimates of neuronal density, roughly 100–120 50 μm^2^ regions were delineated as part of layer 3 or layer 4 in V1 or V2 on NeuN-stained sections from each species, and the number of stained cells in each region was automatically derived using count functions in Ariol. Raw counts were analyzed using the Kolmogorov–Smirnov test and were determined to be non-normal (*p* < 0.0001). Significant differences below *p* = 0.05 were then determined using the Mann–Whitney *U* test in IBM-SPSS (v. 22; IBM, Armonk, NY, USA).

## RESULTS

NeuN and VGLUT2 immunohistochemistry identified neuronal cell bodies and glutamatergic terminations respectively, in V1 of all species examined in this study. Compared to traditional Nissl and CO stains (**Figure 1**), NeuN immunoreactivity (IR) revealed clear laminar boundaries in V1 and V2 (**Figures 2** and **3**), and could distinguish between granule cells and pyramidal cells in all species (**Figure 4**). Eleven distinct laminar divisions in V1 were identified across species and designated as layers or sublayers of the six classic neocortical layers (best seen in **Figure 2**). VGLUT2 IR clearly distinguished V1 layers that receive geniculate inputs, layer 4 and upper layer 6 in all species as well as layer 3Bβ in anthropoid monkeys, and better separated thalamic inputs from intrinsic V1 projections compared to traditional CO stains (**Figure 1**). Additionally, NeuN and VLGUT2 IR revealed the relative shifts of neocortical layers at the boundary of V1 with V2 (**Figures 3** and **5**), providing evidence for a single layer 4 with two subdivisions in V1 that continues as a single undifferentiated layer in V2. The thalamorecipient sublayer of layer 3, 3Bβ, which was previously characterized as part of layer 4 in some species (Brodmann, 1909; Lund, 1988), ended abruptly at the V1/V2 border and did not continue as part of layer 4 in V2. The layers adjacent to layer 4 of V1 however, layers 3C and 5A, did continue across the border from V1 to V2 along with the remaining superficial and deep neocortical layers. A detailed analysis of NeuN and VGLUT2 labeling in each species, as discussed below, provided strong evidence for a uniform laminar scheme in V1 across primates, which can also be applied to highly visual non-primates such as tree shrews.

### LAYERS 1 AND 2 OF V1

Layer 1 of V1 was densely packed with myelinated fibers and largely cell-free in all species (**Figures 1–3, 5**). Scattered populations of small neurons in layer 1 were seen in NeuN preparations across primates, but far fewer neurons were visible in layer 1 of tree shrews (**Figures 1–3**). Layer 2 of V1 contained a thin, dense band of small neurons in all species. In anthropoid monkeys and chimpanzees, layer 2 neurons were more densely packed in the upper half of the layer compared to the lower half, but were more evenly distributed through the layer in prosimian primates and tree shrews (**Figure 2**). In chimpanzees and macaque monkeys, some cells in layer 2 stained weakly for VGLUT2 (**Figures 1** and **5**), but these cells may simply be surrounded by punctate VGLUT2-positive terminals, as seen in other studies of primate VGLUT distributions (Bryant et al., 2012; Garcia-Marin et al., 2012; Balaram et al., 2013). New World monkeys, prosimians, and tree shrews did not express noticeable amounts of VGLUT2 in layer 2.

### LAYER 3 OF V1

Across primates, layer 3 consisted of three distinct sublayers that could be differentiated based on changes in neuronal size and density (**Figures 1–3**). The most superficial layer, 3A, contained heterogeneous populations of medium and large neurons that were concentrated along the upper half of 3A in chimpanzees and macaque monkeys, but evenly distributed across 3A in New World monkeys, prosimians, and tree shrews (**Figure 2**). In chimpanzees and macaque monkeys, some cells in 3A also showed weak labeling for VGLUT2, similar to those seen in layer 2 (**Figures 1** and **5**). Layer 3B, in contrast, mostly contained small neurons closely packed together, and appeared as a cell-dense band between the upper and lower sublayers of layer 3. Neurons in layer 3C were more sparsely distributed compared to neurons in 3A and 3B, giving this layer a lighter appearance in NeuN preparations across species. Both 3B and 3C lacked VGLUT2 labeling in all species, making these layers much lighter in VGLUT2 preparations as well (**Figures 1** and **5**). In general, laminar boundaries between 3A, 3B, and 3C were most easily identified in chimpanzees and macaque monkeys given their clear differences in neuronal density, but were more difficult to distinguish in New World monkeys, prosimians, and tree shrews due to gradual shifts in cell size and density through the depth of layer 3. In prosimians and tree shrews in particular, most of layer 3 appeared to be a continuous population of variably sized cells rather than three identifiable sublayers with distinct variations in neuronal size or density.

At the boundary of layers 3B and 3C in Old World and New World monkeys, a thin band of small granule cells was visible in NeuN preparations (**Figures 1–3**). For macaque, squirrel, and marmoset monkeys, a similar band of dense VGLUT2-positive terminations was present in the same location between 3B and 3C, but in owl monkeys, a more diffuse band of VLGUT2 IR was present instead. This specialized region, termed 3Bβ, receives parvocellular and koniocellular geniculate inputs in anthropoid monkeys (Blasdel and Lund, 1983; Horton, 1984; Lund, 1988), but is less evident in other primates (Casagrande and Kaas, 1994 for review). NeuN preparations through V1 of chimpanzees also showed a similar band of granule-like cells at the border of 3B with 3C, but VGLUT2 preparations did not identify geniculate terminations in this region. Granule-like cells in layer 3 were not seen in prosimians or tree shrews, but all three species did show variations in VGLUT2 labeling in layer 3. V1 in galagos showed diffuse patches of VGLUT2-positive terminals in layer 3B, while V1 in mouse lemurs and tree shrews showed much denser patches of VGLUT2-positive terminations in layer 3 that periodically stretched upward into layers 2 and 1 of V1 (**Figures 1** and **5**). Such variations in VGLUT2 labeling across species may reflect differences in relative parvocellular and koniocellular geniculate inputs to layer 3 of V1.

### LAYER 4 OF V1

Layer 4 is arguably the most distinct layer of V1 in any mammalian species, given its dense array of thalamic visual inputs and its distinct laminar boundaries compared to the superficial and deep layers of V1 (**Figures 1–5**). Across primates, layer 4 consists of two identifiable subdivisions, upper 4A and lower 4B, which receive magnocellular and parvocellular inputs from the LGN, respectively, and send separate projections to other V1 layers (Casagrande and Kaas, 1994; Callaway, 1998). NeuN preparations in all species revealed two subdivisions of layer 4, 4A and 4B, with small, densely distributed granule cells that appeared more crowded together in 4B compared to 4A (**Figures 1–4**). Both divisions were easily identifiable in apes, Old World monkeys and New World monkeys, but were less distinct in prosimians. Interestingly, tree shrews show two similar subdivisions of layer 4, 4A and 4B, but these subdivisions are related to different inputs than those of 4A and 4B in primate species (Conley et al., 1984).

VGLUT2 IR clearly distinguished both subdivisions of layer 4 in all species examined (**Figures 1** and **4**), with 4B showing denser VGLUT2 IR compared to 4A. The extent of VGLUT2 IR in each sublayer was roughly equal in apes and diurnal anthropoid monkeys. In nocturnal primates such as owl monkeys, galagos, and mouse lemurs, however, the magnocellular recipient layer 4A contained a much wider band of VGLUT2 IR compared to the parvocellular recipient layer 4B.

### LAYER 5 OF V1

Layer 5 of V1 is often considered a single layer in primates, although a thin layer of neurons with distinct connections along the dorsal border has been previously observed in macaque monkeys (Lund, 1987). This layer, termed 5A, is not easily distinguished in traditional Nissl stains and has not been previously described in other species. NeuN preparations through V1 clearly identified two sublayers of layer 5 in primates and tree shrews (**Figures 1–3**). The superficial sublayer, 5A, contained small neurons that stained weakly for NeuN, appearing as a thin, pale layer immediately below 4B. The deep sublayer, 5B, contained medium and large neurons that stained darkly for NeuN and were more evenly distributed compared to cells in 5A. The boundary between 5A and 5B was distinguishable in all primates given the distinct change in neuron size and density between the two sublayers. In tree shrews however, the 5A/5B boundary was less distinct due to more variably sized neuronal populations in both sublayers. Nevertheless, two visible subdivisions of layer 5 were present in all examined species.

### LAYER 6 OF V1

Layer 6 of V1 is also traditionally considered a single layer (von Bonin, 1942; Garey, 1971; Lund et al., 1975; Lund, 1988), but tends to have a heterogeneous appearance in most species given its variable cell populations and proximity to the white matter below V1. NeuN preparations in all species identified two sublayers of layer 6; the superficial sublayer, 6A, contained even distributions of medium and large darkly stained neurons while the deep sublayer, 6B, contained sparser populations of small neurons that diffused into the white matter below V1 (**Figures 1–3**). In prosimian primates and tree shrews, a thin cell-free zone often separated cells in 6A from 6B, but in anthropoid primates, 6B appeared continuous with the lower portion of 6A. Layer 6A also contained moderate distributions of VGLUT2 labeling, reflecting sparse geniculostriate inputs to this layer (Lund and Boothe, 1975; Lund, 1988; Casagrande and Kaas, 1994). These projections were more noticeable in New World monkeys compared to other primates but were still visible in all species examined here. In contrast, layer 6B showed no VGLUT2 IR in primates or tree shrews.

### LAMINAR SHIFTS AT THE BOUNDARY OF V1 AND V2

The distinct patterns of lamination seen in V1 of all species ended abruptly at the border of V1 with V2, highlighting the fact that many of the sublayers seen in V1 are specializations of primary visual cortex in primates. When transitioning from V1 to V2, all six neocortical layers shifted slightly toward the pial surface, and superficial cortical layers grew more compact while deep cortical layers expanded in the portion of V2 immediately adjacent to V1 (**Figures 3** and **5**). This dorsal shift occurred over less than a millimeter of cortical surface in most species and, beyond this border region, all layers shifted ventrally in V2, back to similar depths as their corresponding layers in V1. This border region between V1 and V2 contains callosal connections of both areas across primates (Myers, 1962; Wong-Riley, 1974; Essen and Zeki, 1978; Weyand and Swadlow, 1980; Van Essen et al., 1982; Cusick et al., 1984; Innocenti, 1986; Kennedy et al., 1986; Gould et al., 1987; Hof et al., 1997), which may account for the change in relative layer sizes in this region. This shift in neocortical layers was easily visible in chimpanzees and macaque monkeys, and moderately present in New World monkeys and galagos, but only slightly detectable in mouse lemurs. Tree shrews, in contrast, did not show a similar shift in cortical layers along the border of V1 with V2.

The two subdivisions of layer 4 identified in NeuN preparations shifted slightly towards the pial surface when transitioning from V1 to V2 in each case, but both layers continued into V2 as a single, dense layer of granule cells (**Figure 3**). Similarly, the dense VGLUT2 IR seen in layers 4A and 4B of all species also shifted superficially at the V1/ V2 border and merged into a single layer of more moderate VGLUT2 IR in V2 (**Figure 5**). In contrast, the thin band of granule-like cells and VGLUT2-positive terminations in layer 3Bβ of monkeys did not cross the boundary of V1 into V2; in every case, this layer ended abruptly at the V1/V2 boundary, highlighting its specialized existence in some, but not all, primate species. Layer 3C, between layer 3Bβ and 4, did continue from V1 into V2 and could be clearly distinguished as a pale, cell-sparse layer above the densely packed layer 4 in each case. High magnifications of both layer 3C and 4 in V1 and V2 illustrated differences in neuronal size and density between these layers in each area (**Figure 4**). Quantitative analyses of neuronal densities in the middle layers of V1 also showed that neurons in layer 4 were significantly more densely packed than neurons in dorsal layer 3C, across every species examined in this study (*U* = 722487.5, *n* = 1925, *p* < 0.0001). Similar analyses of the middle layers of V2 highlighted the same relationship; layer 4 of V2 contained significantly denser distributions neurons than layer 3C of V2 (*U* = 494858.5, *n* = 1606, *p* < 0.0001) in all species. Such distinct differences between layers 3C and 4 across primates strongly suggests that layer 3C of V1 is not part of layer 4, as originally suggested by Brodmann (1909), but instead derives from a common layer 3 across cortical areas and species, as proposed by Hässler (1967).

## DISCUSSION

The primary goals of this study were to describe patterns of NeuN and VGLUT2 immunoreactivity in V1 of chimpanzees, Old World monkeys, New World monkeys, prosimians, and tree shrews, in order to identify and compare specialized and homologous laminar divisions across primates and closely related non-primate groups. We find that NeuN immunoreactivity identifies a common pattern of lamination in V1 across all these species, regardless of their phyletic origin in the primate lineage. Similarly, VGLUT2 identifies conserved patterns of geniculostriate terminals in layer 4 of all species, as well as specialized patterns of geniculate terminals in layer 3Bβ of anthropoid monkeys, mouse lemurs, and tree shrews. These findings provide anatomical evidence for common laminar architecture in V1, with multiple subdivisions of the superficial and deep layers, but a single layer 4 with two subdivisions, across primate and non-primate species.

Layers 1 and 2 of V1 were clearly homologous across primates and tree shrews, with almost no neurons in layer 1 and small, densely arrayed neurons in layer 2. The faint VGLUT2 IR in layer 2 of chimpanzees and macaque monkeys likely derives from pulvinar projections to layer 2 of V1 (Balaram et al., 2013; Marion et al., 2013) that may also exist in New World monkeys (Tigges et al., 1981; Stepniewska and Kaas, 1997; Soares et al., 2001; Kaas and Lyon, 2007) and prosimians (Raczkowski and Diamond, 1980; Wong and Kaas, 2010). The three subdivisions of layer 3 seen in NeuN preparations also appear to be highly conserved across primates, and are likely related to similar divisions of layer 3 in non-primates as well. The clustered arrangement of medium and large pyramidal neurons in 3A is morphologically similar across primates and tree shrews, as is the dense distribution of smaller pyramids in layer 3B. Both layers appear expanded in anthropoid primates compared to those in prosimians and tree shrews, and this may reflect the greater number of total neurons in V1 of anthropoid compared to prosimian primates (Collins et al., 2010).

Layer 3Bβ appears to be unique to anthropoid monkeys, given the lack of dense CO reactivity or VGLUT2-positive terminations in layer 3 of chimpanzees (Preuss et al., 1999; Bryant et al., 2012), and humans (Bryant et al., 2012; Garcia-Marin et al., 2012). In apes and humans, thalamic inputs to layer 3B of V1 appear to be reduced or absent altogether (Preuss et al., 1999; Preuss and Coleman, 2002; Bryant et al., 2012; Garcia-Marin et al., 2012). The presence of VGLUT2- positive geniculate terminations in layer 3B of tree shrews and mouse lemurs, however, raises the possibility that parvocellular and koniocellular inputs to this region arose early in the ancestors of the arboreal primate lineage. In nocturnal galagos and owl monkeys, where VGLUT2-positive terminations were more diffuse, parvocellular projections are reduced and layer 3 is dominated by koniocellular inputs instead. Thus, parvocellular inputs to layer 3Bβ appear to have evolved early on in the primate lineage, and they were either refined to a single layer in diurnal, arboreal anthropoid monkeys or they gradually receded in nocturnal or terrestrial primates such as galagos, owl monkeys, chimpanzees and humans.

In all primates, koniocellular geniculate terminations are often coincident with the well-known CO blobs of V1 (Livingstone and Hubel, 1982; Horton, 1984; Lachica and Casagrande, 1992; Casagrande et al., 2007), but the colocalization of VGLUT2 IR with CO blobs is not well documented across primates and not all koniocellular geniculate projections utilize VGLUT2 (Balaram et al., 2011, 2013). In prosimians and tree shrews, VGLUT2 IR does identify patchy blob-like structures in layer 3B of V1 that correspond to CO-dense blobs or patches in the same layer (Wong and Kaas, 2009b, 2010). However, in New World and Old World monkeys, as well as humans, VGLUT2 IR only reveals sparse distributions of geniculate terminals in layer 3B that may be periodic and coincident with the CO blobs (Bryant et al., 2012; Garcia-Marin et al., 2012; Balaram et al., 2013). These differences in VGLUT2-positive terminations between prosimians, anthropoid primates, and humans suggest that geniculate terminations to the blob regions of V1 were more diffuse in ancestral primates, were refined to sparser inputs in anthropoid monkeys, and may be further reduced in present-day chimpanzees and humans.

The termination of VGLUT2-positive projections to 3Bβ at the V1/V2 border in most species does suggest that this layer is unique to V1. Although it has been common to conclude that layers 3Bβ and 4 of V1 merge to form a single layer in V2, a close examination of the border region between V1 and V2 in NeuN and VGLUT2 preparations shows that this is not the case. The slight dorsal shift of all cortical layers at the V1/V2 boundary may give the illusion of merging middle layers from V1 to V2, but only one granular layer continues uninterrupted from V1 to V2 in NeuN preparations of each species examined here. Similarly, only one layer of dense VGLUT2-positive terminations continues from V1 to V2 in each species, the single layer 4 as previously delineated by Hässler (1967). Further evidence for this conclusion comes from the clear identification of layer 3C in V1, which also continues uninterrupted into V2. The presence of large pyramidal neurons instead of small granule cells, as well as the lack of VGLUT2-positive geniculate projections, differentiates this layer from the standard definition of layer 4 - a single layer characterized by small granule cells that receive dense inputs from the LGN (Cowey, 1964; Hubel and Wiesel, 1972; Dräger, 1974; Hubel et al., 1975; Ribak and Peters, 1975; Glendenning et al., 1976; Kaas et al., 1976; LeVay and Gilbert, 1976; Cooper et al., 1979; Spatz, 1979; Towns et al., 1982; Rosa et al., 1996). Neurons in layer 3C instead receive intrinsic projections from layer 4, similar to 3A and 3B (Levitt et al., 1996), and are known to project extrinsically to MT (Van Essen et al., 1981; Maunsell and Van Essen, 1983; Shipp and Zeki, 1989) and the thick CO bands of V2 (Federer et al., 2009; Sincich et al., 2010), just as 3A and 3B project extrinsically to V2 and other visual areas (DeYoe and Van Essen, 1985; Rockland, 1992; Sincich and Horton, 2005; Sincich et al., 2010; Federer et al., 2013). Analyses of neuronal density in layers 3C and 4 of V1 and V2 showed that both layers are quite distinct from one another, regardless of the area in question. Other studies on laminar specific gene expression in macaque monkeys show that neurons in Hässler’s 3Bβ and 3C are genetically more similar to layer 3 neurons than layer 4 neurons across multiple areas in the neocortex. Thus, Brodmann’s delineation of this layer as part of layer 4 seems inappropriate given the evidence discussed above. Hässler’s (1967) laminar scheme for V1 however, allows for better comparisons of V1 morphology and function across all primates, as well as tree shrews and other visual mammals.

Layer 4 across primates and tree shrews could be identified by small, densely packed granule cells in NeuN preparations as well as dense VGLUT2 IR in both subdivisions, 4A and 4B. Both sublayers merged with a single layer 4 containing similarly sized granule cells but more moderate VGLUT2 labeling in V2. This layer 4, which corresponds to Hässler’s layer 4 of V1, is directly comparable to layer 4 of V1 in rodents (Krieg, 1946; Peters and Feldman, 1976; Caviness and Frost, 1980; Frost and Caviness, 1980; Simmons et al., 1982; Peters, 1985), lagomorphs (Swadlow and Weyand, 1981; Towns et al., 1982; Weyand and Swadlow, 1986), and lemurs (Clark, 1931; Zilles et al., 1979; Preuss and Kaas, 1996), tarsiers (Collins et al., 2005), and great apes and humans (Yoshioka and Hendry, 1995; Preuss et al., 1999; Preuss and Coleman, 2002; Zilles et al., 2002; Preuss, 2007; Bryant et al., 2012). By excluding the variable sublayer 3Bβ with koniocellular LGN inputs as well as the pyramidal sublayer 3C with no thalamic inputs, layer 4 can be consistently identified as a single granular layer across mammals, thus creating a more unified scheme of V1 lamination across primate and non-primate species.

Layer 5 of V1 surprisingly revealed two consistent subdivisions across species, which have only been previously reported in macaque monkeys (Lund, 1973, 1987; Lund et al., 1988; Balaram et al., 2013). The superficial sublayer, 5A, consists largely of interneurons that project intrinsically in V1, and are identifiable in the present results by comparatively weak NeuN labeling against the surrounding V1 layers. In contrast, the deep sublayer 5B could be differentiated by stronger NeuN IR and sparser distributions of medium and large pyramidal neurons, which are known to project subcortically to the pulvinar and superior colliculus in most primate species (Spatz et al., 1970; Lund et al., 1975; Graham et al., 1979; Raczkowski and Diamond, 1980; Baldwin and Kaas, 2012; Baldwin et al., 2013). The consistent identification of two sublayers in layer 5 across primates and tree shrews suggests that these patterns of intrinsic and extrinsic projections are highly conserved in these closely related species. Further studies in non-primate species will reveal if these sublayers are fundamental to V1 organization across other mammals as well.

Similarly, layer 6 of V1 has been recently subdivided into two layers across multiple primate and non-primate species, largely based on distinct differences in gene expression patterns between cells in the upper portion, 6A, and cells in the lower portion, 6B (Yamamori and Rockland, 2006; Hevner, 2007; Belgard et al., 2011; Bernard et al., 2012). The present results also reveal differences in cellular density between the two layers, as well as the presence of geniculate terminations throughout 6A but not 6B. Prior reports of V1 lamination largely consider layer 6 as a single layer that blends into the ventral white matter (Lund, 1973; Lund et al., 1988; Casagrande and Kaas, 1994), but subtle differences between upper and lower tier cells in this layer have been considered (Levitt et al., 1996; Yamamori and Rockland, 2006; Watakabe et al., 2012, 2006). Our results, in combination with recent studies of layer- and cellular-specific gene expression, suggest that layer 6 is made up of two functional subdivisions; a superficial sublayer with sparse geniculate inputs as well as intrinsic and extrinsic visual projections, and a deep sublayer with separate inputs and functions. Both sublayers are consistently present across primates and tree shrews, and are similar to those seen in rodent species as well (Usrey and Fitzpatrick, 1996; Hevner, 2007; Belgard et al., 2011; Bernard et al., 2012).

The results presented here contribute to a unified understanding of laminar organization in V1 across multiple primates as well as a closely related non-primate, tree shrews. They highlight the inconsistencies of Brodmann’s laminar scheme when comparing V1 architecture across multiple species and augment the growing number of reports that promote the use of Hässler’s laminar scheme in V1 research (Spatz et al., 1970; Spatz, 1977; Tigges and Tigges, 1981; Tigges et al., 1982; Maunsell and Van Essen, 1983; Gould et al., 1987; Allman and McGuinness, 1988; Henry, 1989; Casagrande and Kaas, 1994). In conjunction with reports of physiological and connectional differences between V1 layers, they will drive further research on the homologous and specialized laminar features of primate V1.

## AUTHOR CONTRIBUTIONS

Conception, data acquisition, analysis, interpretation, drafting of manuscript, final review and approval, accountability – Pooja Balaram and Jon H. Kaas.

## Conflict of Interest Statement

The authors declare that the research was conducted in the absence of any commercial or financial relationships that could be construed as a potential conflict of interest.
